# Serum neurofilament light chain levels in migraine patients: a monocentric case–control study in China

**DOI:** 10.1186/s10194-023-01674-2

**Published:** 2023-11-06

**Authors:** Jie Fang, Jielong Wu, Tengkun Zhang, Xiaodong Yuan, Jiedong Zhao, Liangcheng Zheng, Ganji Hong, Lu Yu, Qing Lin, Xingkai An, Chuya Jing, Qiuhong Zhang, Chen Wang, Zhanxiang Wang, Qilin Ma

**Affiliations:** 1 Department of Neurology and Department of Neuroscience, The First Affiliated Hospital of Xiamen University, School of Medicine, Xiamen University, 55 Zhenhai Road, Xiamen, 361003 China; 2 The School of Clinical Medicine, Fujian Medical University, Fuzhou, China; 3 Fujian Key Laboratory of Brain Tumors Diagnosis and Precision Treatment, Xiamen, China; 4 Xiamen Key Laboratory of Brain Center, Xiamen, China; 5 Xiamen Medical Quality Control Center for Neurology, Xiamen, China; 6 Fujian Provincial Clinical Research Center for Brain Diseases, Xiamen, China; 7 Xiamen Clinical Research Center for Neurological Diseases, Xiamen, China; 8 School of Medicine, Xiamen University, Xiamen, China; 9 National Institute for Data Science in Health and Medicine, Xiamen University, Xiamen, China; 10 Department of Neurology, The Fifth Hospital of Xiamen, Xiamen, China; 11 Department of Gynecology, Xiamen Maternal and Child Health Care Hospital, Xiamen, China; 12 Cerebrovascular Interventional Department, Zhangzhou Hospital of Fujian Province, Zhangzhou, China; 13 Department of Neurology, Changxing People’s Hospital, Huzhou, China; 14 Department of Neurosurgery and Department of Neuroscience, the First Affiliated Hospital of Xiamen University, School of Medicine, Xiamen University, 55 Zhenhai Road, Xiamen, 361003 China

**Keywords:** Serum neurofilament light chain, Migraine, Migraine disease course, Neurological damage

## Abstract

**Purpose:**

Serum neurofilament light chain (sNfL) can reflect nerve damage. Whether migraine can cause neurological damage remain unclear. This study assesses sNfL levels in migraine patients and explores whether there is nerve damage in migraine.

**Methods:**

A case–control study was conducted in Xiamen, China. A total of 138 migraine patients and 70 healthy controls were recruited. sNfL (pg/mL) was measured on the single-molecule array platform. Univariate, Pearson correlation and linear regression analysis were used to assess the relationship between migraine and sNfL levels, with further subgroup analysis by migraine characteristics.

**Results:**

Overall, 85.10% of the 208 subjects were female, with a median age of 36 years. sNfL levels were higher in the migraine group than in the control group (4.85 (3.49, 6.62) vs. 4.11 (3.22, 5.59)), but the difference was not significant (*P* = 0.133). The two groups showed an almost consistent trend in which sNfL levels increased significantly with age. Subgroup analysis showed a significant increase in sNfL levels in patients with a migraine course ≥ 10 years (β = 0.693 (0.168, 1.220), *P* = 0.010). Regression analysis results show that age and migraine course are independent risk factors for elevated sNfL levels, and there is an interaction between the two factors. Patients aged < 45 years and with a migraine course ≥ 10 years have significantly increased sNfL levels.

**Conclusions:**

This is the first study to evaluate sNfL levels in migraine patients. The sNfL levels significantly increased in patients with a migraine course ≥ 10 years. More attention to nerve damage in young patients with a long course of migraine is required.

**Supplementary Information:**

The online version contains supplementary material available at 10.1186/s10194-023-01674-2.

## Background

Migraine, a common chronic disorder of the nervous system, afflicts approximately 1 billion people worldwide [1, 2]. Its basic clinical feature is recurrent moderate to severe headaches that last from 4 to 72 h [3, 4]. The pathogenesis is not fully understood, and it is generally believed to be related to the trigeminal vascular system [5, 6].

In the past, migraine was thought to be a benign disorder that did not cause neurological damage. However, recent studies have revealed serological evidence of neurological damage in migraine patients. S100 calcium-binding protein B (S100B) and neuron-specific enolase (NSE), as biomarkers of central nervous system damage, have been widely detected in several types of migraine [7, 8]. The combined results of nine case–control studies indicated that compared with healthy controls, overall migraine patients had significantly increased S100B levels in peripheral blood (SMD = 0.688, 95% CI: 0.341–1.036, *P* < 0.001) [7]. In contrast, a case–control study indicated that interictal serum S100B levels are not elevated in migraine patients [9]. In a study on pediatric migraine, serum NSE levels did not change during migraine attacks [10]. Therefore, the use of NSE and S100B as biomarkers for determining the presence of neurological damage in migraine remains to be further investigated. It would be interesting to find other biomarkers to assist in the determination.

Neurofilament light chain (NfL) is enriched in large myelinated axons, playing a role in stabilizing the cytoskeleton and accelerating signal transduction [11–13]. Serum neurofilament light chain (sNfL), as a biomarker reflecting neurological damage, has been measured in various neurological diseases, such as Parkinson's, multiple sclerosis and amyotrophic lateral sclerosis, and has played a corresponding role in assessing disease severity and determining prognosis [14].

Although sNfL has been measured extensively in other neurological disorders, there are still few studies on this marker in the field of migraine. As axonal injury is the pathological basis of disability in various neurological disorders, quantifying this injury by measuring sNfL levels is important for assessing disease activity, monitoring treatment response, and evaluating prognosis. Therefore, in this study, we used a single-molecule array to assess sNfL levels in migraine patients versus healthy controls and explores whether there is nerve damage in migraine.

## Methods

### Study design and participants

A case–control study was conducted in Xiamen, China. A total of 138 migraine patients who visited the First Affiliated Hospital of Xiamen University between January 2020 and December 2021 were selected for inclusion in the case group. In addition, 70 healthy volunteers were included as a control group. The inclusion criteria for the case group were as follows: (1) migraine diagnosed according to the ICHD-3; (2) not having received prophylactic treatment; (3) absence of headache within 72 h; and (4) absence of serious systemic diseases, including tumors, autoimmune diseases, thyroid diseases, etc.; and (5) absence of other chronic neurological disorders, such as Parkinson's, multiple sclerosis, Wilson's disease and epilepsy; (6) no previous history of traumatic brain injury or cardiovascular system disease. The inclusion criteria for the control group were as follows: (1) no history of primary or secondary headache; and items (4), (5), and (6) mentioned above.

### Clinical characteristics

Age and sex of healthy control subjects were collected. Detailed medical history information was collected from all enrolled migraine patients, including sex, age (years), migraine course (years), migraine duration (hours), migraine frequency (days/per month), family history of migraine, pain location, presence or absence of aura, accompanying symptoms (nausea, vomiting, photophobia, phonophobia), disability, menstrually related migraine, and days since last migraine attack. Consistent with other studies, migraine duration was classified as follows: ‘12 h’ (reference group) and ‘ > 12 h’. Migraine frequency was classified as follows: ‘4 days per month’ (reference group),’5–8 days per month’, and ‘9–15 days per month’. All of the above variables were used as covariates in the regression analyses.

### Quantification of sNfL

The key factor of concern is the sNfL level (pg/mL). All study subjects had 5 ml of blood drawn from the antecubital vein, and blood samples of migraine patients were collected during the headache-free period. Blood samples were centrifuged at 3000 rpm for 8 min, and the supernatant was stored at -80 °C until the NfL assay was completed. Blood sNfL concentrations were measured with the single molecule array platform provided by Quanterix. Measurements were performed on the fully automated instrument HD-1 Analyzer (Quanterix) using the NF-L kit from Quanterix, which employs two anti-NfL monoclonal antibodies produced by Quanterix.

### Statistical analysis

First, descriptive statistics for participants’ characteristics were tabulated. Pearson's chi-squared test, and Wilcoxon rank sum test were used to compare the differences between groups. Second, scatter plots and Pearson correlation coefficients were used to examine the linear associations between age and sNfL levels in the control group and migraine group, respectively. Third, after controlling for gender and age, multivariate linear regression was used to evaluate the impact of migraine on sNfL levels, with further subgroup analysis by migraine characteristics. Fourth, we simultaneously consider the impact of age and migraine course on patients' sNfL levels, and analyze their interaction effect. We performed multivariable linear regressions of sNfL on the main associations of the combined age, migraine course and cross-product interaction terms (Model1-3). Model 1 analysis was adjusted for gender to account for potential demographic confounders. Model 2 analysis was additionally adjusted for migraine duration and migraine frequency. Model 3 analysis was adjusted for all the covariates mentioned previously (including sex, migraine duration, migraine frequency, family history of migraine, pain location, presence or absence of aura, accompanying symptoms, disability, menstrually related migraine, and days since last migraine attack). Furthermore, since there is interaction effect between age and migraine course, we stratified patients by their age and migraine course to examine the joint effect of age and migraine course and to identify the migraine patients with the highest sNfL levels. Adjusting for sex and age, beta coefficient and corresponding 95% confidence intervals (CIs) were estimated accordingly, with healthy controls being the reference category. Last, we performed further exploratory subgroup analyses of the young migraineurs group with ≥ 10 year of migraine course. And the results were visualized using violin plots, box plots and scatter plots. All statistical calculations were performed using R (version 4.2.1, 2022 R Foundation for Statistical Computing), *p* values less than 0.05 were considered to indicate statistical significance.

## Results

### Characteristics of participants

The 208 subjects included 70 healthy controls and 138 migraineurs. Overall, 85.10% of the 208 subjects were female, with a median (IQR) age of 36.00 (29.00, 45.00). There was no significant difference in the gender distribution between the two groups, and the migraine group was older than the control group (37.00 (31.00, 45.00) vs. 33.00 (27.00, 43.75)), but the difference was not significant (*p* = 0.053). sNfL levels were higher in the migraine group than in the control group (4.85 (3.49, 6.62) vs. 4.11 (3.22, 5.59)), but the difference was not significant (*P* = 0.133). Among migraine patients, the median (IQR) migraine course was 7.00 (3.00, 15.00) years. For more details, see Table 1.

**Table 1:** Clinicodemographic characteristics and sNfL levels of study participants

Characteristic	Total			
	**Overall, *N* = 208**^a^	**Control, *N* = 70**^a^	**Migraine, *N* = 138**^a^	***P***^b^
Sex				0.518
Male	31 (14.90%)	12 (17.14%)	19 (13.77%)	
Female	177 (85.10%)	58 (82.86%)	119 (86.23%)	
Age (years)	36.00 (29.00, 45.00)	33.00 (27.00, 43.75)	37.00 (31.00, 45.00)	0.053
Migraine course (years)		NA	7.00 (3.00, 15.00)	
Migraine duration (hours)		NA	10.00 (4.25, 20.00)	
Migraine frequency (days/per month)		NA	2.00 (1.00, 6.00)	
Family history of migraine		NA	86 (62.32%)	
Migraine with aura		NA	31 (22.46%)	
Bilateral pain location		NA	37 (26.81%)	
Nausea		NA	113 (81.88%)	
Vomiting		NA	83 (60.14%)	
Photophobia		NA	60 (43.48%)	
Phonophobia		NA	53 (38.41%)	
Any accompanying symptoms		NA	122 (88.41%)	
Disability		NA	58 (42.03%)	
Menstrually related migraine		NA	13 (9.42%)	
Days since last migraine attack		NA	7.00 (6.00, 12.00)	
sNfL (pg/mL)	4.75 (3.44, 6.37)	4.11 (3.22, 5.59)	4.85 (3.49, 6.62)	0.133

^a^n (%), Median (IQR), ^b^Pearson's Chi-squared test, Wilcoxon rank sum test

### Age and sNfL levels

**Fig. 1:**
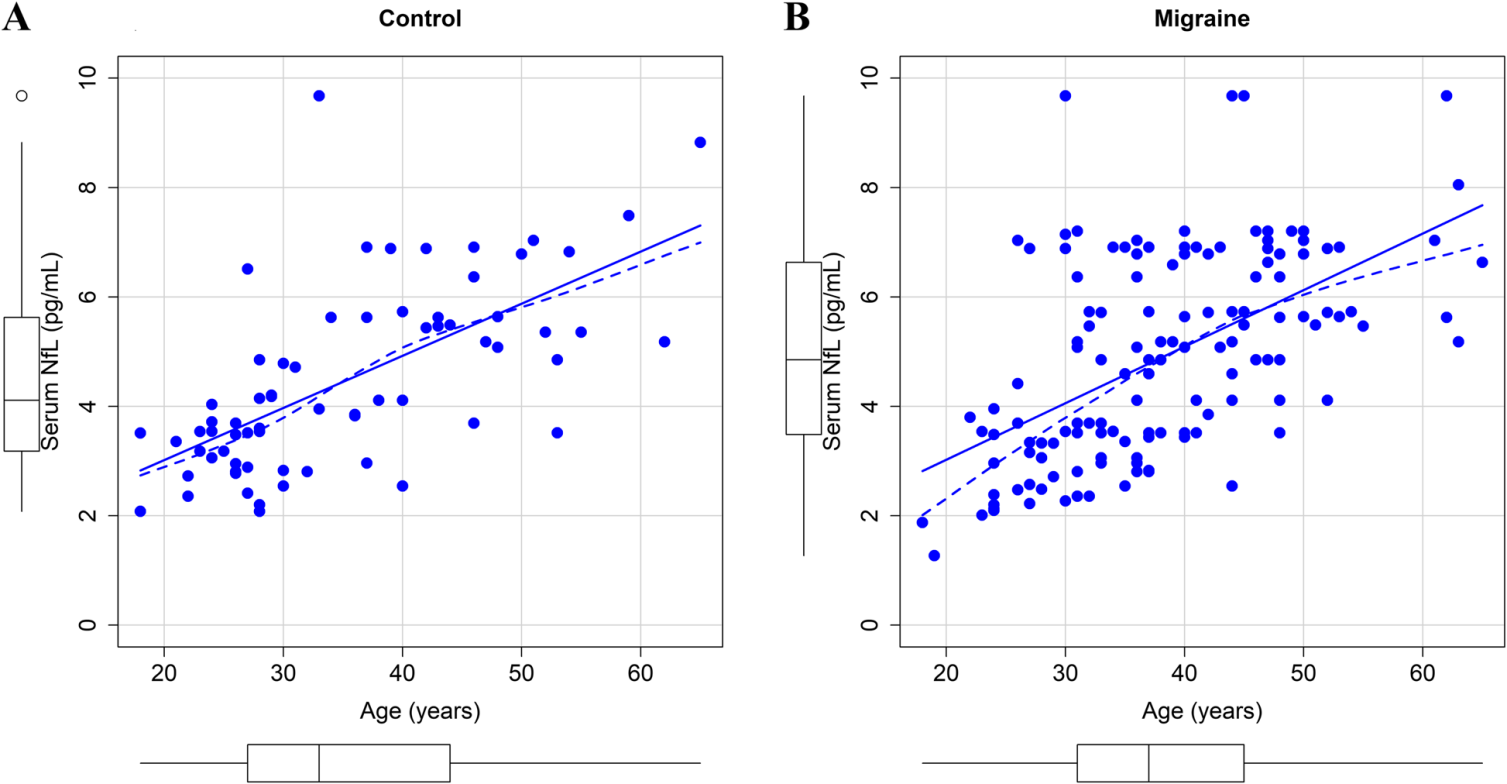
Correlation between serum neurofilament light chain (sNfL) levels and age. (**A**) in healthy controls group (**B**) in migraine group

As shown in Fig. 1, in the healthy control group, the older the subject was, the higher the sNfL level (*r* = 0.648 (0.488, 0.767), *P* < 0.05). Similarly, in the migraine group, the older the patient was, the higher the sNfL level (*r* = 0.558 (0.431, 0.663), *P* < 0.05). The two groups showed an almost consistent trend in which sNfL levels increased significantly with age.

### The impact of migraine on sNfL levels

The association between migraine and sNfL levels is illustrated in Table 2. On the whole, after control for sex and age, the migraine was not significantly associated with sNfL levels (β = 0.142 (-0.283, 0.566), *P* = 0.512). In the subgroup analysis results, compared with healthy control, there was a significant increase in sNfL levels in patients with a migraine course ≥ 10 years (β = 0.693 (0.168, 1.220), *P* = 0.010). However, in other subgroups, no significant increase in sNfL levels was found in migraine patients.

**Table 2:** The relationship between migraine and sNfL levels

**Characteristic**	**Category**	**N (%)**	**β**^*a*^	**95% CI**^*b*^	***P***
Migraine		138 (100.00%)	0.142	-0.283, 0.566	0.512
**Subgroup Analysis**					
Migraine course (years)	< 10	76 (55.07%)	-0.18	-0.624, 0.265	0.426
	10	62 (44.93%)	**0.693**	**0.168, 1.22**	**0.01**
Migraine duration (hours)	12	89 (64.49%)	-0.005	-0.433, 0.423	0.983
	> 12	49 (35.51%)	0.421	-0.136, 0.98	0.137
Migraine frequency (days/per month)	4	94 (68.12%)	0.158	-0.307, 0.622	0.504
	5–8	25 (18.12%)	0.028	-0.588, 0.645	0.927
	9–15	19 (13.77%)	0.211	-0.471, 0.894	0.54
Family history of migraine	No	52 (37.68%)	0.252	-0.269, 0.774	0.34
	Yes	86 (62.32%)	0.081	-0.372, 0.534	0.724
Migraine with aura	No	107 (77.54%)	0.152	-0.274, 0.579	0.481
	Yes	31 (22.46%)	0.129	-0.499, 0.758	0.684
Bilateral pain location	No	101 (73.19%)	0.142	-0.293, 0.578	0.519
	Yes	37 (26.81%)	0.152	-0.432, 0.736	0.607
Any accompanying symptoms	No	16 (11.59%)	-0.342	-1.08, 0.400	0.362
	Yes	122 (88.41%)	0.202	-0.229, 0.633	0.357
Disability	No	80 (57.97%)	0.101	-0.380, 0.583	0.678
	Yes	58 (42.03%)	0.211	-0.262, 0.684	0.38
Menstrually-related migraine	No	125 (90.58%)	0.137	-0.295, 0.569	0.532
	Yes	13 (9.42%)	0.113	-0.685, 0.911	0.779

^a^β coefficient for association with sNfL, with healthy controls being the reference category, ^b^*CI* Confidence Interval. Estimated were adjusted for sex and age

### Interaction of age with migraine course

In Table 3, we show the Regression of age, migraine course and their cross-product term on sNfL levels by adjusting for different covariates (Model 1–3). Regression analysis results show that age and migraine course are independent risk factors for elevated sNfL levels, and there is an interaction between the two factors (*P* < 0.001). In the result of Model 3 (adjusting for sex and different migraine characteristics), the estimated effect size of age on migraine patients’ sNfL is 0.119 (0.080, 0.159), and the estimated effect size of migraine course on migraine patients’ sNfL is 0.315 (0.197, 0.432). The effect of migraine course on sNfL varies with the age (interaction term’ *P* < 0.001).

**Table 3:** Regression of age, migraine course and their cross-product term on sNfL levels^a^

Characteristic	Model 1		Model 2		Model 3	
	**β (95% CI)**	***P***	**β (95% CI)**	***P***	**β (95% CI)**	***P***
Age (years)	0.118 (0.081, 0.156)	< 0.001	0.119 (0.081, 0.156)	< 0.001	0.119 (0.080, 0.159)	< 0.001
Migraine course (years)	0.298 (0.186, 0.409)	< 0.001	0.302 (0.189, 0.414)	< 0.001	0.315 (0.197, 0.432)	< 0.001
Age × Migraine course^b^	-0.005 (-0.007, -0.003)	< 0.001	-0.005 (-0.007, -0.003)	< 0.001	-0.005 (-0.008, -0.003)	< 0.001

^a^β (95% CI): beta coefficient and corresponding 95% confidence intervals. Model 1 analysis was adjusted for gender. Model 2 analysis was additionally adjusted for migraine duration and migraine frequency. Model 3 analysis was additionally adjusted for other migraine characteristics

^b^*P* for interaction

**Fig. 2:**
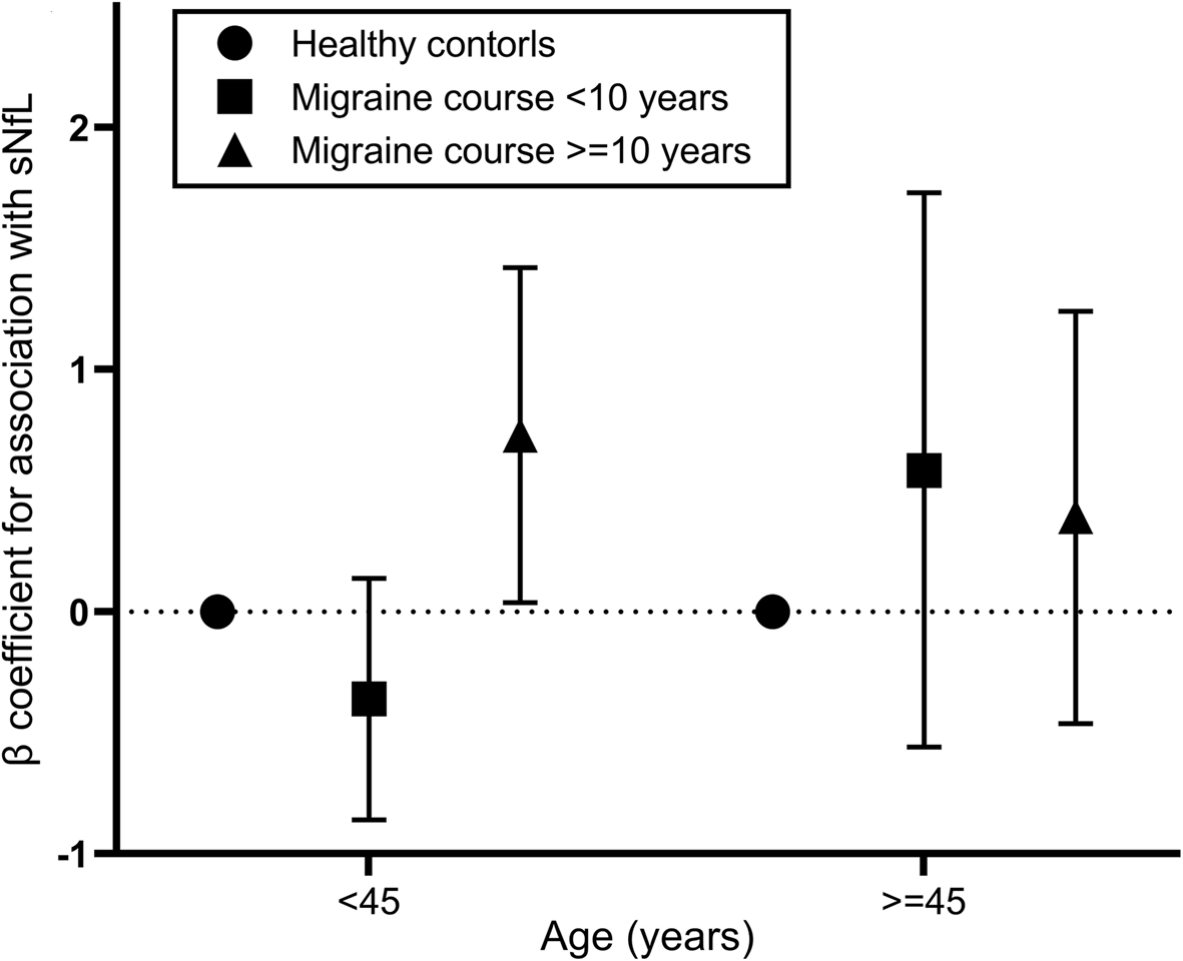
Joint associations of age and migraine course. The symbols indicate point estimates of β and the vertical bars indicate the corresponding 95% CIs. Healthy controls were the reference categories. Estimated were adjusted for sex

The association of combination of age and migraine course are shown in Fig. 2. Longer migraine course was associated with higher sNfL levels among young patients, but no such trend was observed for patients who age  45 years. Compared with healthy control groups, patients aged < 45 years and with a migraine course ≥ 10 years have significantly increased sNfL levels (β = 0.728 (0.038, 1.420), *P* = 0.039).

### Exploratory subgroup analyses

Supplementary Fig. 1 demonstrates the sNfL levels in different subgroups in the young migraineurs group with ≥ 10 years of disease duration (*N* = 32). There was no significant difference in sNfL levels in patients with different gender, migraine duration, migraine frequency, family history of migraine, migraine with aura, bilateral pain location, nausea, vomiting, photophobia, phonophobia, disability, and days since last migraine attack were not significantly associated with sNfL levels in the young migraineurs group with ≥ 10 years of disease duration (*P* > 0.05).

## Discussion

In this case–control study, using data from 138 migraine patients and 70 healthy controls, we demonstrate a significant elevation in migraine patients’ sNfL levels as age and migraine course progresses. Patients aged < 45 years and with a migraine course ≥ 10 years have significantly increased sNfL levels. This suggests that migraine can cause nerve damage. To our knowledge, this is the first study to evaluate nerve damage in migraine disease by sNfL levels.

In the study, comparing the 138 migraine patients with 70 healthy controls, we were unable to find a difference in sNfL levels between the two groups, either by univariate or multivariate regression analysis (after controlling for gender and age). This may be due to differences in the distribution of other characteristics between the two groups. It has been suggested that body mass index is also an important factor in sNfL levels [15]. There are also significant differences in NfL levels in subjects with other comorbidities, such as diabetes, depression, anxiety, sleep disorders and cognitive impairment [16–19]. In addition, the median disease duration (IQR) of the migraine patients included in this study was 7.00 (3.00, 15.00) years, and the short duration of disease in most patients may have led to a nonsignificant difference between the migraine and healthy groups. In the subgroup analysis results, compared with healthy control, there was a significant increase in sNfL levels in patients with a migraine course ≥ 10 years (β = 0.693 (0.168, 1.220), *P* = 0.010). A recent study by Overeem et al. found that after adjusting for covariates of age, gender, and BMI, no statistically significant differences were observed in sNfL between the healthy control group, episodic migraine, and chronic migraine (H = 1.359, *P* = 0.507) [20]. Before conducting subgroup analysis on the course of migraine, we obtained exactly the same results as Overeem's study when adjusting for covariates of age, gender (β = 0.142 (-0.283, 0.566), *P* = 0.512). However, that study did not report the course of migraine, and we cannot determine whether the effect of migraine course on sNfL remains consistent across different populations.

Consistent with previous studies, we observed a significant increase in sNfL levels with age. Both healthy controls and migraine patients showed an almost consistent trend in which sNfL levels increased significantly with age. Besides, in an effort to identify the influence factors of migraine patients’ sNfL levels, the association of the separation and interaction effect of age and migraine course with sNfL were assessed, when adjusting for sex, migraine duration, migraine frequency, family history of migraine, pain location, presence or absence of aura, accompanying symptoms, disability, menstrually related migraine, and days since last migraine attack as potential confounders. With age rising and the migraine course prolongs, the patient's sNfL levels significantly increase. Based on the theoretical basis that elevated sNfL levels can reflect axonal damage, we speculate that migraine may be a completely reversible disease in the early stages of the disease, and nerve damage may not occur at this stage. However, as the course of migraine prolongs, nerve damage may gradually occur.

Regression analysis results also show that there is an interaction between age and migraine course. The effect of migraine course on sNfL varies with the migraine course. To clarify this interaction effect on sNfL, we further explored the associations of the combination of age and migraine course in relation to sNfL. Since the incidence rate and disease burden of migraine in young people is significantly higher [4, 21, 22], our study divided the population into two groups (age < 45 years and ≥ 45 years) to explore the impact of migraine course on sNfL levels in different age groups. Longer migraine course was associated with higher sNfL levels among young patients, but no such trend was observed for patients who age  45 years. The elderly population is often accompanied by various chronic diseases and frailty conditions [23, 24], which may mask the effect of migraine course on sNfL. A recent study indicates that sNfL concentrations rose exponentially with age and at a steeper increased rate after approximately 50 years of age [25]. In current study, we found that patients aged < 45 years and with a migraine course ≥ 10 years have significantly increased sNfL levels. It suggested that more attention to nerve damage in young patients with a long course of migraine is required.

The question of whether migraine causes nerve damage has remained inconclusive in former studies. Previous researchers have used the S100B protein and NSE as markers to try to understand whether nerve damage is present in migraine but have had conflicting results [7–9]. Recent studies have identified changes in grey matter volume and white matter abnormalities in migraine patients by magnetic resonance imaging (MRI) [26–28]. In multiple sclerosis and multiple system atrophy (MSA), it has been shown that there is a strong correlation between NfL levels and MRI analysis results [29, 30]. Therefore, it is reasonable to suspect that NfL levels, as real-time, easily measured markers of response to axonal injury, have potential clinical application in migraine.

Gaetani et al. describe the level of sNfL elevation compared to healthy controls in various central nervous system diseases in their review article [31]. Consistent with the levels that are measured patients with neurodegenerative diseases like Alzheimer’s disease (it includes both prodromal AD and dementia due to AD), Parkinson’s disease, and Parkinson’s disease dementia, our study found that the mean fold increase of NfL in the young migraineurs group with  10 year of migraine history was less than 2. Although the mean fold increase of NfL is relatively small, studies have shown that sNfL levels can serve as a predictive indicator of cognitive stage transition [16]. This therefore reminds us that sNfL levels may serve as an effective biomarker for response to severity of the disease and different stages of migraine.

Although direct evidence for a correlation between migraine and sNfL levels is lacking from previous studies, we can find clues from the association between migraine and other neurological disorders. An observational study from Wuhan, China, found a significant correlation between migraine duration and cognitive dysfunction, with both migraine duration and frequency of attacks affecting cognitive function [32]. The prevalence of migraine was significantly higher in patients with multiple sclerosis than in normal subjects [33]. There is growing evidence that migraine increases the overall risk of developing cerebrovascular disease, including stroke [34]. At the same time, sNfL has been shown to be a reliable biomarker for cognitive dysfunction, multiple sclerosis, stroke, and other diseases [14, 16, 35–37]. The potential link between migraine and these diseases may explain the variation in sNfL levels over the course of the disease in migraineurs.

In the current study, we did not find an effect of migraine characteristics such as migraine duration, attack frequency, or concomitant symptoms on sNfL levels. Although we observed higher sNfL levels in patients with migraine durations > 12 h, the differences were not statistically significant. A larger sample size needs to be collected to further evaluate the effect of indicators such as headache duration on sNfL levels. On the other hand, the headache profile of migraine is related to a variety of factors, and headache symptoms are modulated by a variety of factors and are not invariable [38–40]. The relationship between migraine symptoms and sNfL levels needs to be discussed in the context of the changing migraine profile of patients. Although there is currently no effective medication that can completely cure migraine and shorten the migraine course [41], it may be possible to prevent nerve damage by reducing the duration of headaches and other intervention methods.

There are still some limitations to this study. First, the subjects included in this study were all from the same hospital, which limits the extrapolation of the findings. Second, although this study observed a correlation between migraine course and sNfL levels, further longitudinal follow-up data are appropriate to observe the characteristics of the study population over time and to analyze the sources of heterogeneity between individuals. Third, there is possible confounding bias. In exploring differences in sNfL levels between the migraine group and healthy controls, only gender and age were controlled for, without controlling for additional influencing factors (e.g., other chronic conditions). Due to the exclusion of migraine patients using preventive treatment, the impact of preventive and therapeutic drugs on sNfL levels in migraine patients was not explored. The use of migraine medication may influence patients' sNfL levels while improving migraine symptoms. Again, more patient characteristics (e.g., body mass index, diet, etc.) could have been included in the study to effectively control for bias.

## Conclusions

In summary, this study explored sNfL levels in migraine patients for the first time and found a correlation between the migraine course and sNfL levels, especially in young patients. The findings provide new evidence that migraine can cause nerve damage and contribute to the understanding of migraine. More attention to nerve damage in young patients with a long course of migraine is required. And more studies are needed in the future to further validate the clinical application of sNfL levels as a biomarker for migraine disease.

### Supplementary Information

**Additional file 1:** **Supplementary Figure 1.** The sNfL levels in different subgroups in the young migraineurs group with >10 years of disease duration. Legend: Each point in the graph represents the characteristics of a patient. *P***-**values were estimated using the Wilcoxon rank sum test, Independent-Samples Kruskal–Wallis Test, and Pearson correlation analysis, respectively.

## Data Availability

The data supporting the study results may be accessed via the corresponding author upon reasonable request.

## Abbreviations

sNfL: Serum neurofilament light chain
S100B: S100 calcium-binding protein B
NSE: Neuron-specific enolase
MRI: Magnetic resonance imaging
MSA: Multiple system atrophy

## References

[1] Ashina M, Katsarava Z, Do TP, Buse DC, Pozo-Rosich P, Ozge A, Krymchantowski AV, Lebedeva ER, Ravishankar K, Yu S (2021). Migraine: epidemiology and systems of care. Lancet.

[2] (2017) Nations within a nation: variations in epidemiological transition across the states of India 1990–2016 in the Global Burden of Disease Study. The Lancet 390(10111):2437–60. 10.1016/S0140-6736(17)32804-0 10.1016/S0140-6736(17)32804-0 PMC5720596 29150201

[3] Eigenbrodt AK, Ashina H, Khan S, Diener HC, Mitsikostas DD, Sinclair AJ, Pozo-Rosich P, Martelletti P, Ducros A, Lanteri-Minet M (2021). Diagnosis and management of migraine in ten steps. Nat Rev Neurol.

[4] (2018) Headache Classification Committee of the International Headache Society (IHS). The International Classification of Headache Disorders, 3rd edn. Cephalalgia 38(1):1–211. 10.1177/0333102417738202 10.1177/0333102417738202 29368949

[5] Burstein R, Noseda R, Borsook D (2015). Migraine: multiple processes, complex pathophysiology. J Neurosci.

[6] Zhang X, Levy D, Kainz V, Noseda R, Jakubowski M, Burstein R (2011). Activation of central trigeminovascular neurons by cortical spreading depression. Ann Neurol.

[7] Chu C, Zhong R, Cai M, Li N, Lin W (2022). Elevated Blood S100B Levels in Patients With Migraine: A Systematic Review and Meta-Analysis. Front Neurol.

[8] Gonen M, Ozdogan S, Balgetir F, Demir CF, Aytac E, Mungen B (2021). S100B and neuron-specific enolase levels in episodic and chronic migraine. Acta Neurol Scand.

[9] Riesco N, Cernuda-Morollon E, Martinez-Camblor P, Perez-Pereda S, Pascual J (2020). Peripheral, Interictal Serum S100B Levels are Not Increased in Chronic Migraine Patients. Headache.

[10] Azapagasi E, Alehan F, Saygi S, Bayraktar N, Yazici AC (2012). Serum concentrations of neuron-specific enolase in pediatric migraine. Turk J Pediatr.

[11] Gafson AR, Barthélemy NR, Bomont P, Carare RO, Durham HD, Julien JP, Kuhle J, Leppert D, Nixon RA, Weller RO (2020). Neurofilaments: neurobiological foundations for biomarker applications. Brain.

[12] Bomont P (2021). The dazzling rise of neurofilaments: Physiological functions and roles as biomarkers. Curr Opin Cell Biol.

[13] Sainio MT, Rasila T, Molchanova SM, Järvilehto J, Torregrosa-Muñumer R, Harjuhaahto S, Pennonen J, Huber N, Herukka SK, Haapasalo A (2021). Neurofilament light regulates axon caliber, synaptic activity, and organelle trafficking in cultured human motor neurons. Front Cell Dev Biol.

[14] Khalil M, Teunissen CE, Otto M, Piehl F, Sormani MP, Gattringer T, Barro C, Kappos L, Comabella M, Fazekas F (2018). Neurofilaments as biomarkers in neurological disorders. Nat Rev Neurol.

[15] Koini M, Pirpamer L, Hofer E, Buchmann A, Pinter D, Ropele S, Enzinger C, Benkert P, Leppert D, Kuhle J (2021). Factors influencing serum neurofilament light chain levels in normal aging. Aging (Albany NY).

[16] Lee EH, Kwon HS, Koh SH, Choi SH, Jin JH, Jeong JH, Jang JW, Park KW, Kim EJ, Kim HJ (2022). Serum neurofilament light chain level as a predictor of cognitive stage transition. Alzheimers Res Ther.

[17] Urso D, Batzu L, Logroscino G, Ray Chaudhuri K, Pereira JB (2023). Neurofilament light predicts worse nonmotor symptoms and depression in Parkinson's disease. Neurobiol Dis.

[18] Li Y, Li F, Liu X, Zu J, Zhang W, Zhou S, Zhu J, Zhang T, Cui G, Xu C (2023). Association between serum neurofilament light chain levels and sleep disorders in patients with Parkinson's disease. Neurosci Lett.

[19] Maalmi H, Strom A, Petrera A, Hauck SM, Strassburger K, Kuss O, Zaharia OP, Bönhof GJ, Rathmann W, Trenkamp S (2023). Serum neurofilament light chain: a novel biomarker for early diabetic sensorimotor polyneuropathy. Diabetologia.

[20] Lucas H, Overeem B, Raffaelli R, Fleischmann M, Süße A, Vogelgesang AM, et al (2023) Serum tau protein elevation in migraine: a cross-sectional case–control study. Abstr J Headache Pain 24(1) 10.1186/s10194-023-01663-5 10.1186/s10194-023-01663-5 PMC10507851 37726712

[21] Ferrari MD, Goadsby PJ, Burstein R, Kurth T, Ayata C, Charles A, Ashina M, van den Maagdenberg A, Dodick DW (2022). Migraine Nat Rev Dis Primers.

[22] (2017) Global regional and national incidence prevalence and years lived with disability for 328 diseases and injuries for 195 countries 1990–2016: a systematic analysis for the Global Burden of Disease Study 2016. The Lancet 390(10100);1211–59. 10.1016/S0140-6736(17)32154-2 10.1016/S0140-6736(17)32154-2 PMC5605509 28919117

[23] Chuang YN, Chen CC, Wang CJ, Chang YS, Liu YH (2023). Frailty and polypharmacy in the community-dwelling elderly with multiple chronic diseases. Psychogeriatrics.

[24] Tang H, Tyler K, Chan P (2023). Frailty status and related factors in elderly patients in intensive care for acute conditions in China. Am J Health Behav.

[25] Benkert P, Meier S, Schaedelin S, Manouchehrinia A, Yaldizli Ö, Maceski A, Oechtering J, Achtnichts L, Conen D, Derfuss T (2022). Serum neurofilament light chain for individual prognostication of disease activity in people with multiple sclerosis: a retrospective modelling and validation study. Lancet Neurol.

[26] Chong CD, Schwedt TJ, Trivedi M, Chong BW (2022). The Characteristics of White Matter Hyperintensities in Patients With Migraine. Front Pain Res (Lausanne).

[27] Laukka D, Parkkola R, Hirvonen J, Ylikotila P, Vahlberg T, Salo E, Kivelev J, Rinne J, Rahi M (2022). Brain white matter hyperintensities in Kawasaki disease: a case-control study. Front Neurosci.

[28] Chen ZH, Cui YL, Sun JT, Li YT, Zhang C, Zhang YM, Li ZY, Shang YX, Ni MH, Hu B (2022). The brain structure and function abnormalities of migraineurs: a systematic review and neuroimaging meta-analysis. Front Neurol.

[29] Barro C, Benkert P, Disanto G, Tsagkas C, Amann M, Naegelin Y, Leppert D, Gobbi C, Granziera C, Yaldizli O (2018). Serum neurofilament as a predictor of disease worsening and brain and spinal cord atrophy in multiple sclerosis. Brain.

[30] (2022) Neurofilament light levels predict clinical progression and death in multiple system atrophy. Abstract Brain 145(12):4398–4408. 10.1093/brain/awac253 10.1093/brain/awac253 PMC9762941 35903017

[31] Gaetani L, Blennow K, Calabresi P, Di Filippo M, Parnetti L, Zetterberg H (2019). Neurofilament light chain as a biomarker in neurological disorders. J Neurol Neurosurg Psychiatry.

[32] Huang L, Juan Dong H, Wang X, Wang Y, Xiao Z (2017). Duration and frequency of migraines affect cognitive function: evidence from neuropsychological tests and event-related potentials. J Headache Pain.

[33] Kister I, Caminero AB, Monteith TS, Soliman A, Bacon TE, Bacon JH, Kalina JT, Inglese M, Herbert J, Lipton RB (2010). Migraine is comorbid with multiple sclerosis and associated with a more symptomatic MS course. J Headache Pain.

[34] Oie LR, Kurth T, Gulati S, Dodick DW (2020). Migraine and risk of stroke. J Neurol Neurosurg Psychiatry.

[35] De Marchis GM, Katan M, Barro C, Fladt J, Traenka C, Seiffge DJ, Hert L, Gensicke H, Disanto G, Sutter R (2018). Serum neurofilament light chain in patients with acute cerebrovascular events. Eur J Neurol.

[36] Zhao Y, Xin Y, Meng S, He Z, Hu W (2019). Neurofilament light chain protein in neurodegenerative dementia: A systematic review and network meta-analysis. Neurosci Biobehav Rev.

[37] Sellebjerg F, Magyari M (2022). The prognostic value of neurofilament light chain in serum. Lancet Neurol.

[38] Scher AI, Buse DC, Fanning KM, Kelly AM, Franznick DA, Adams AM, Lipton RB (2017). Comorbid pain and migraine chronicity: the chronic migraine epidemiology and outcomes study. Neurology.

[39] Vetvik KG, MacGregor EA (2017). Sex differences in the epidemiology, clinical features, and pathophysiology of migraine. Lancet Neurol.

[40] Messina R, Cetta I, Colombo B, Filippi M (2022). Tracking the evolution of non-headache symptoms through the migraine attack. J Headache Pain.

[41] Ezzati A, Buse DC, Fanning KM, Reed ML, Martin VT, Lipton RB (2022). Predictors of treatment-response to acute prescription medications in migraine: Results from the American Migraine Prevalence and Prevention (AMPP) Study. Clin Neurol Neurosurg.

