# Supplementary figures and images for: Serum neurofilament light chain levels in migraine patients: a monocentric case–control study in China
Source: J Headache Pain. 2023 Nov 6;24(1):149. doi: 10.1186/s10194-023-01674-2 (PMC10626745; doi:10.1186/s10194-023-01674-2)

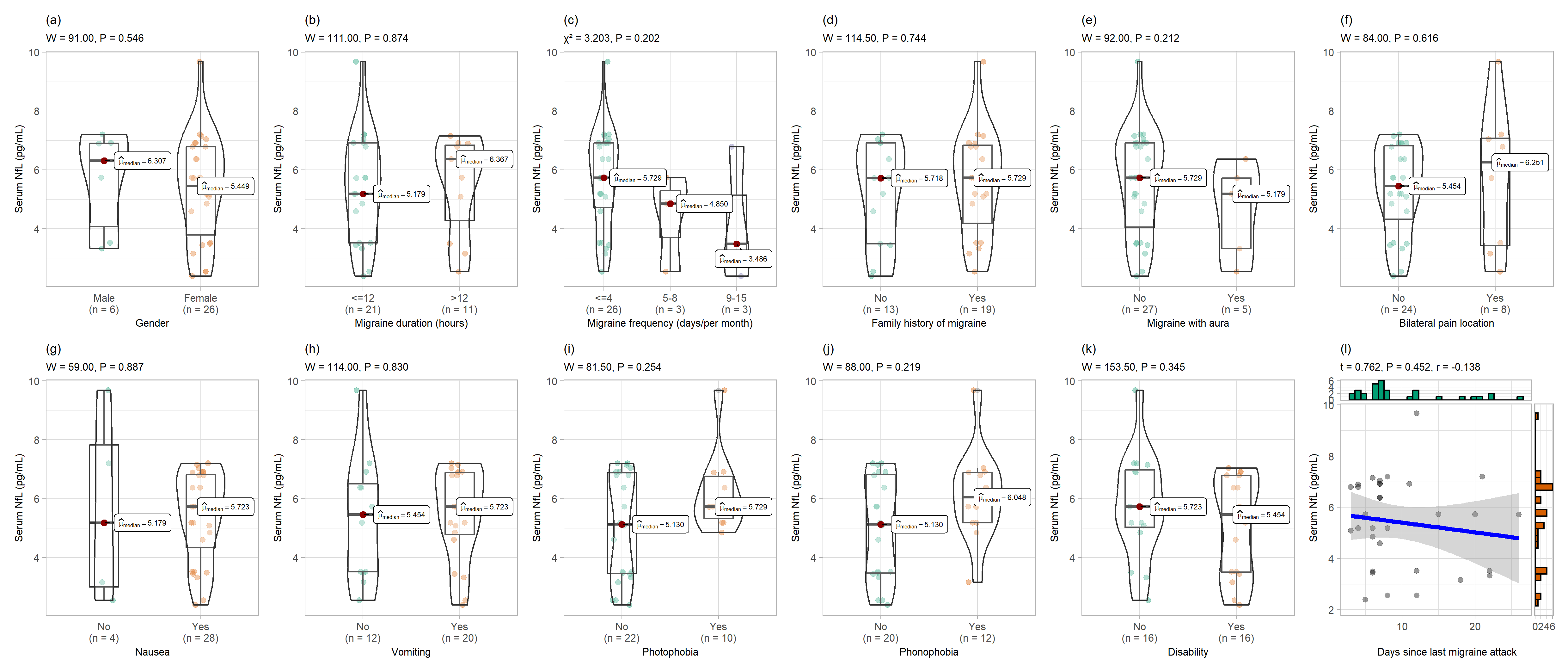

Supplement: Supplementary file 1 — Additional file 1: Supplementary Figure 1. The sNfL levels in different subgroups in the young migraineurs group with >10 years of disease duration. Legend: Each point in the graph represents the characteristics of a patient. P-values were estimated using the Wilcoxon rank sum test, Independent-Samples Kruskal–Wallis Test, and Pearson correlation analysis, respectively. [file 10194_2023_1674_MOESM1_ESM.tif]
